# Functional Analysis of the *Arabidopsis thaliana* CDPK-Related Kinase Family: At*CRK1* Regulates Responses to Continuous Light

**DOI:** 10.3390/ijms19051282

**Published:** 2018-04-25

**Authors:** Abu Imran Baba, Gábor Rigó, Ferhan Ayaydin, Ateeq Ur Rehman, Norbert Andrási, Laura Zsigmond, Ildikó Valkai, János Urbancsok, Imre Vass, Taras Pasternak, Klaus Palme, László Szabados, Ágnes Cséplő

**Affiliations:** 1 Plant Biology Institute, Biological Research Centre, Hungarian Academy of Sciences, 6726 Szeged, Hungary; baba.abuimran@brc.mta.hu (A.I.B.); ayaydin.ferhan@brc.mta.hu (F.A.); rehman.ateequr@brc.mta.hu (A.U.R.); andrasi.norbert@brc.mta.hu (N.A.); zsigmond.laura@brc.mta.hu (L.Z.); valkai.ildiko@brc.mta.hu (I.V.); vass.imre@brc.mta.hu (I.V.); szabados.laszlo@brc.mta.hu (L.S.); 2 Doctoral School in Biology, Faculty of Science and Informatics, University of Szeged, 6720 Szeged, Hungary; 3 Department of Plant Biology, University of Szeged, 6726 Szeged, Hungary; 4 Department of Biology, Norwegian University of Science and Technology, Høgskoleringen 5, NO-7491 Trondheim, Norway; janos.urbancsok@ntnu.no; 5 Faculty of Biologie II, Albert-Ludwigs Universität, Schänzlestr. 1, 79104 Freiburg, Germany; taras.p.pasternak@gmail.com (T.P.); klaus.palme@biologie.uni-freiburg.de (K.P.)

**Keywords:** CDPK-related kinase (CRK) family, plasma membrane localization, gravitropic response, singlet oxygen, cell death, *Arabidopsis thaliana*

## Abstract

The Calcium-Dependent Protein Kinase (CDPK)-Related Kinase family (CRKs) consists of eight members in *Arabidopsis*. Recently, At*CRK5* was shown to play a direct role in the regulation of root gravitropic response involving polar auxin transport (PAT). However, limited information is available about the function of the other At*CRK* genes. Here, we report a comparative analysis of the *Arabidopsis* CRK genes, including transcription regulation, intracellular localization, and biological function. At*CRK* transcripts were detectable in all organs tested and a considerable variation in transcript levels was detected among them. Most AtCRK proteins localized at the plasma membrane as revealed by microscopic analysis of 35S::cCRK-GFP (Green Fluorescence Protein) expressing plants or protoplasts. Interestingly, 35S::cCRK1-GFP and 35S::cCRK7-GFP had a dual localization pattern which was associated with plasma membrane and endomembrane structures, as well. Analysis of T-DNA insertion mutants revealed that At*CRK* genes are important for root growth and control of gravitropic responses in roots and hypocotyls. While At*crk* mutants were indistinguishable from wild type plants in short days, At*crk1-1* mutant had serious growth defects under continuous illumination. Semi-dwarf phenotype of At*crk1-1* was accompanied with chlorophyll depletion, disturbed photosynthesis, accumulation of singlet oxygen, and enhanced cell death in photosynthetic tissues. At*CRK1* is therefore important to maintain cellular homeostasis during continuous illumination.

## 1. Introduction

Changes in environmental conditions represent a continuous stimulation for plants, which need adequate responses in metabolic adjustment, growth, and development. Plants possess an intricate signaling system, which senses stimuli, transmits signals, and coordinates responses in transcriptional, translational, metabolic, and developmental levels. Signal transduction has numerous players, which includes reactive oxygen species (ROS), changes in cellular Ca^2+^ level, lipid signals and a range of posttranslational protein modifications including phosphorylation, myristoylation, and sumoylation. Transcription regulation is the usual target of cellular signaling and modulation of gene expression is achieved through activation or repression of particular sets of transcription factors.

Various abiotic and biotic stimuli can cause changes in cellular Ca^2+^ levels in plant cells [1,2,3,4,5,6,7,8,9,10,11]. The Calcium-Dependent Protein Kinases (CDPKs) are the main regulators in Ca^2+^ signaling [1,2,3,4,12]. CDPKs and the Ca^2+^/Calmodulin-Dependent Protein Kinase-Related Kinases (CRKs) are Ser/Thr protein kinases, which have regulatory functions in diverse processes like plant growth and development, abiotic and biotic stress responses [1,2,3,4,5,13] as well as in phytohormone regulation [6]. So far, CRKs have been identified only in angiosperms. A unique feature of the CDPKs/CRKs superfamily is their N-terminal myristoylation site, suggesting that these proteins are localized at the plasma membrane or other endomembrane system [6,7,14,15,16,17,18]. The CDPKs have a variable N-terminal, a catalytic, an autoinhibitory domain, and a calmodulin-like (CaM) domain at their C-terminus. The latter domain (EF-hand) of CDPKs is able to bind calcium mediating environmental effects to downstream signals [2,3,4,5]. Unlike CDPKs, CRKs have degenerated EF-hand motifs at their *C*-termini, which are unable to bind Ca^2+^ directly [14]. CRKs have highly variable N-terminal sequences, except for two conserved domains, similar to a myristoylation/palmitoylation motif and a putative nuclear localization signal [13,15]. Mutations in N-terminal Gly-2 and Cys-4 positions lead to cytoplasmic and nuclear localization of tomato (*Solanum lycopersicum*) CRK1 [16], indicating the importance of the myristoylation in plasma membrane localization [15,17,18]. All known CRKs share a C-terminal calmodulin (CaM) binding domain, which overlaps with the kinase autoinhibitory domain [13,15]. In CRKs, the selective calmodulin binding in the presence of Ca^2+^ stimulates autophosphorylation but leads to only a slight increase of substrate phosphorylation [14,15,16,19].

While abundant information is available about the CDPK and SnRK kinases [3,4,5,6,7,8,9,10,13], the biological function of plant CRK subfamily is less known [2,15,20,21,22,23]. In tomato, the LeCRK1 was suggested to participate in fruit ripening [16]. The tomato CRK family consists of six members, one of them (SlCRK6) was implicated in disease resistance [4]. The importance of the CDPK/CRK genes in the regulation of latex production was recently proved in the rubber tree *Hevea brasiliensis* [24].

In *Arabidopsis* sp., the CRK subfamily is structurally closely related to CDPKs and consists of eight members [1,2]. However, only a few of them have been characterized. AtCRK1 (also mentioned as At*CBK3*) was reported to bind calmodulin (CaM) in a Ca^2+^-dependent manner, while autophosphorylation and substrate phosphorylation activity was found to be independent of Ca^2+^ [21]. AtCRK1/AtCBK3 interacts with and phosphorylates AtHSFA1a and is a positive regulator of heat shock responses [25] and regulator of salt sensitivity [23]. AtCRK3 is known to interact with the cytosolic glutamine synthetase AtGLN1;1 and postulated to be involved in leaf senescence [26]. Three *Arabidopsis* CRKs—AtCRK2, AtCRK3, and AtCRK8—were able to phosphorylate the C-terminal tyrosine (Tyr) residues of β-tubulin (TBB) 2 and TBB7, the major constituent of microtubules. Moreover, AtCRK2 and AtCRK3 had high in vitro Tyr-phosphorylation activity for certain transcription factors like ethylene response factor 13 (ERF13), WRKY DNA-binding protein 14 (WRKY14), ERF subfamily B-4 of ERF/AP2 transcription factor 2.6 (RAP2.6), and cryptochrome-interacting basic helix-loop-helix5 (CIB5). Moreover, AtCRKs, in addition to their Ser/Thr phosphorylation capability, were claimed to have Tyr kinase activity, assuming their roles in Tyr phosphorylation signaling in angiosperm [27]. AtCRK5 was shown to have a direct role in the regulation of root gravitropic response and polar auxin transport by phosphorylating the PIN2 auxin transport protein, suggesting that AtCRKs are potential regulators of responses to different environmental stimuli [15]. To reveal information on their biological function, we carried out a systematic characterization of other AtCRK members. We determined their intracellular localization through microscopic study of 35S::cCRK-eGFP gene fusions and cell fractionation. Biological functions were characterized by analysis of T-DNA insertion knockout or knockdown mutants. We show, that most AtCRK genes influence root growth, root and hypocotyl gravitropism, while At*CRK1* is implicated in responses to continuous light and cellular redox homeostasis.

## 2. Results

### 2.1. Variation of CRK Genes in Arabidopsis sp.

**Figure 1:**
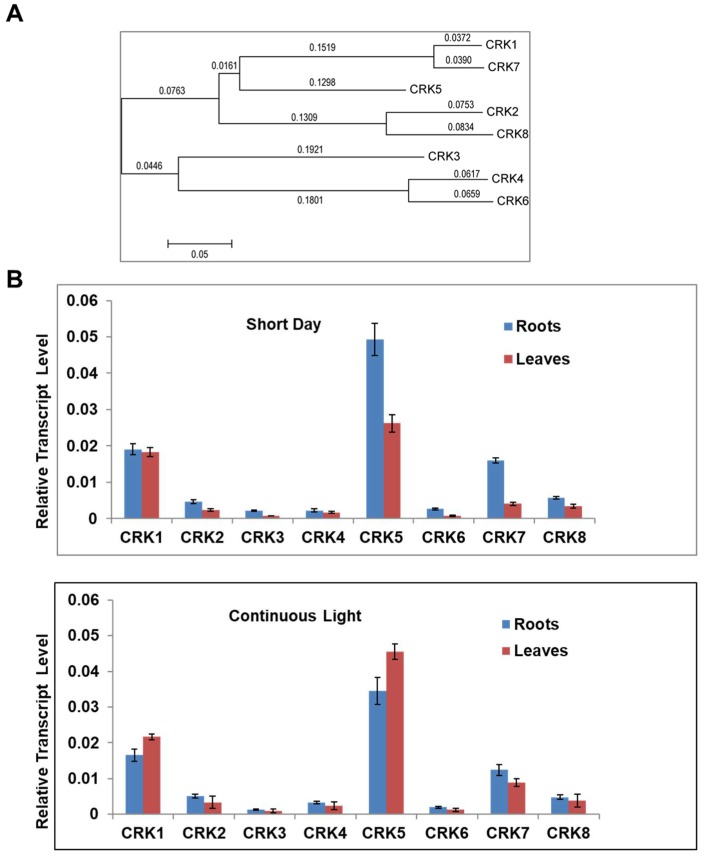
Relationship and expression of the AtCRK genes. (**A**) Phylogenetic tree of AtCRK proteins, using MEGA5.05 software, http://www.megasoftware.net/software. Bootstrap values are indicated. During phylogeny reconstruction, we used a neighbor-joining bootstrap method with 500 bootstrap replications; (**B**) AtCRK expression in wild type *Arabidopsis* sp. plants. Transcript levels were determined in the rosette leaves and roots of 14 days old wild type *Arabidopsis* sp. plants at short day and continuous light conditions using qRT-PCR. Relative transcript levels were standardized to GAPDH-2 (At1g13440). Reactions were made in three replicates. Bars indicate standard deviation (SD) carried out with two biological repetitions.

Annotation of the *Arabidopsis* genome revealed the existence of eight CDPK-related kinase (At*CRK1*-At*CRK8*) genes, which encode proteins with a high degree of sequence similarity. A phylogenetic tree showed pairwise close relationships between AtCRK1 and AtCRK7, AtCRK4 and AtCRK6, and AtCRK2 and AtCRK8 proteins (Figure 1A).

Analysis of public transcript profiling data (eFP Browser, Table S1) [28] suggests that there is a considerable difference between the expression levels and organ specificities of these genes. Among the eight genes tested, At*CRK5* had the highest transcript levels, while At*CRK6* expression was on the limit of detection. While expression of At*CRK5* was particularly high in flowers, transcript abundances of the other genes was rather variable (Table S1). According to eFP Browser data, external stimuli had only moderate effects on the expression of most AtCRK genes. Light had no strong effect on AtCRKs expression, although expression of At*CRK1*, At*CRK3,* and At*CRK5* was slightly enhanced, while At*CRK2*, At*CRK4,* and At*CRK7* were reduced by light. When transcript levels were tested in rosettes and roots of in vitro grown two-week old plantlets cultured under short day and continuous light conditions, AtC*RK5* and At*CRK1* had the highest, while At*CRK3* and At*CRK6* displayed the lowest expression levels (Figure 1B). The length of illumination had an undetectable or only slight influence on the transcript levels of the AtCRK genes tested.

### 2.2. Intracellular Localization of AtCRK Proteins

To compare intracellular localization of the individual AtCRK kinases, full length cDNAs were fused in-frame to the coding sequence of green fluorescent protein (eGFP) and overexpressed in *Arabidopsis* sp. cells under the control of the CaMV35S promoter of the pK7FWG2.0 expression vector [29]. AtCRK5 was previously shown to be associated with the plasma membrane [15]. Transient expression of the gene fusions in *Arabidopsis* sp. protoplasts showed a membrane-bound localization pattern for all AtCRK proteins, similar to AtCRK5 (Figure S1). The only exception was AtCRK1, which showed a different pattern of localization in the cytosol, possibly associated to the endomembrane system.

**Figure 2:**
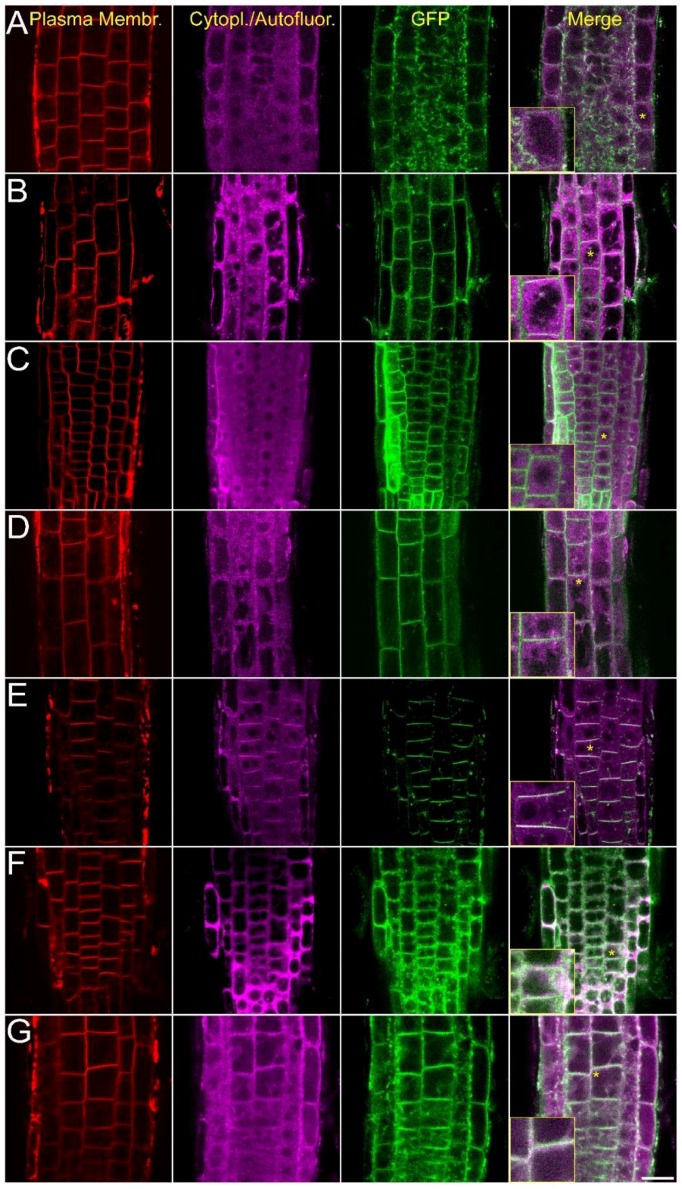
Intracellular localization of the AtCRK-eGFP fusion proteins. AtCRK protein localization in root cells of transgenic *Arabidopsis* plants expressing the pCaMV35S::CRK-eGFP gene constructs. FM4-64 dye labeling indicates plasma membranes (red, first column). Violet laser induced (excitation: 405 nm) cytoplasmic autofluorescence is shown in the second column (pseudocolored in magenta, detected between 425–475 nm). GFP-conjugated CRK protein images (green, CRK1 to CRK8) are merged with autofluorescence images at the last column where vacuoles appear as dark intracellular areas due to lack of autofluorescence and absence of CRKs. Yellow asterisks indicate regions from which 2× magnified closeup images (insets) are prepared. (**A**) AtCRK1-GFP; (**B**) AtCRK2-GFP; (**C**) AtCRK3-GFP; (**D**) AtCRK4-GFP; (**E**) AtCRK5-GFP; (**F**) AtCRK7-GFP; (**G**) AtCRK8-GFP. Bar = 20 µm.

To test the intracellular localization of AtCRK proteins in intact tissues, the roots of transgenic plants expressing the 35S::cCRK-GFP constructs were investigated by confocal laser scanning microscopy (CLSM). In meristematic zone of roots, GFP-derived fluorescence was associated to the perimeters of the cells and colocalized with plasma membrane-specific FM4-64 stain, indicating plasma membrane localization (Figure 2). Fluorescence of the 35S::cCRK1-GFP and 35S::cCRK7-GFP fusions were slightly diffused in meristematic cells of roots assuming an additional endomembrane location of these proteins.

**Figure 3:**
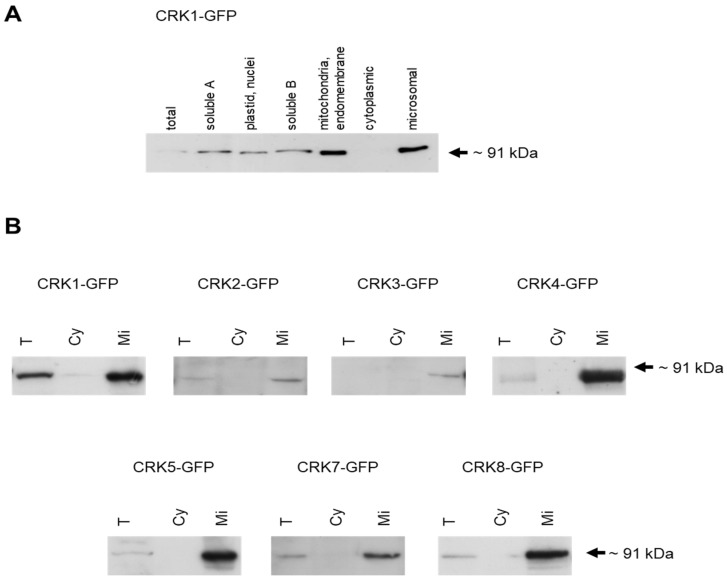
Distribution of AtCRK-GFP proteins in subcellular fractions. Cellular extracts were separated by differential centrifugation and CRK-eGFP was detected by Western hybridization with an antibody recognizing GFP (anti-GFP). Equal amounts of protein (25 µg) were loaded in each lane. (**A**) Identification of AtCRK1-eGFP in six cellular fractions, separated by differential centrifugation; (**B**) Distribution of AtCRK1-eGFP, AtCRK2-eGFP, AtCRK3-eGFP, AtCRK4-eGFP, AtCRK7-eGFP, and AtCRK8-eGFP in two cellular fractions. All AtCRK-eGFP proteins were detected in the microsomal fractions and but not in the cytoplasmic soluble fractions. T: total protein extract, Cy: cytoplasmic soluble, Mi: microsomal membrane fraction.

Differential centrifugation of plant extracts followed by immunoblotting revealed that AtCRK-GFP proteins were present in the microsomal membrane fractions (Figure 3). The observed microsomal membrane enrichment of these AtCRK proteins correlates with the presence of N-terminal myristoylation sites [15,17]. In case of the CRK1-GFP protein, the strongest signals could be detected both in the mitochondria/endomembrane and microsomal fractions (Figure 3A). All the other AtCRK-GFP were found in microsomal membrane fraction (Figure 3B). The weak AtCRK2-GFP and AtCRK3-GFP signal in the microsomal fraction is probably the consequence of protein degradation during protein isolation.

Intracellular spotty localization of 35S::cCRK1-GFP protein resembles mitochondria or Golgi localization. High resolution spinning disc confocal microscopy analyses combined with organelle markers showed that these structures are not related to mitochondria (Mitotracker Orange labeling, Figure S2D–F) or Golgi apparatus (Golgi-specific mCherry expression, Figure S2G–I). Screening online protein/dye localization databases to find a similar localization pattern of a protein or an organelle was not fruitful. Therefore, the exact nature of these bulbous interconnected membranous structures remaines to be identified.

### 2.3. Function of AtCRK Genes, Analysis of T-DNA Insertion Mutants

For functional analysis, we identified T-DNA insertion mutations for all AtCRK family members from public mutant collections. Presence of T-DNA insertions could be confirmed in most but not all lines by PCR, using combination of T-DNA specific and gene specific primers (Table S2). Putative knockout or knockdown mutants could be identified for each AtCRK genes, except for At*CRK6* and At*CRK7*, for which we were unable to identify mutants. Expression of the AtCRK genes in the homozygous mutant lines was tested by quantitative RT-PCR. At*crk1-1*, At*crk2-1*, At*crk2-6*, At*crk3-1,* and At*crk-8-1* proved to be real knockout (KO) mutants. At*crk1-3*, At*crk4-1*, At*crk4-2* were found to be knockdown (KD) mutants to different degrees (Figure S3). The At*crk5-1* mutant was already published to be KO mutant [15].

**Figure 4:**
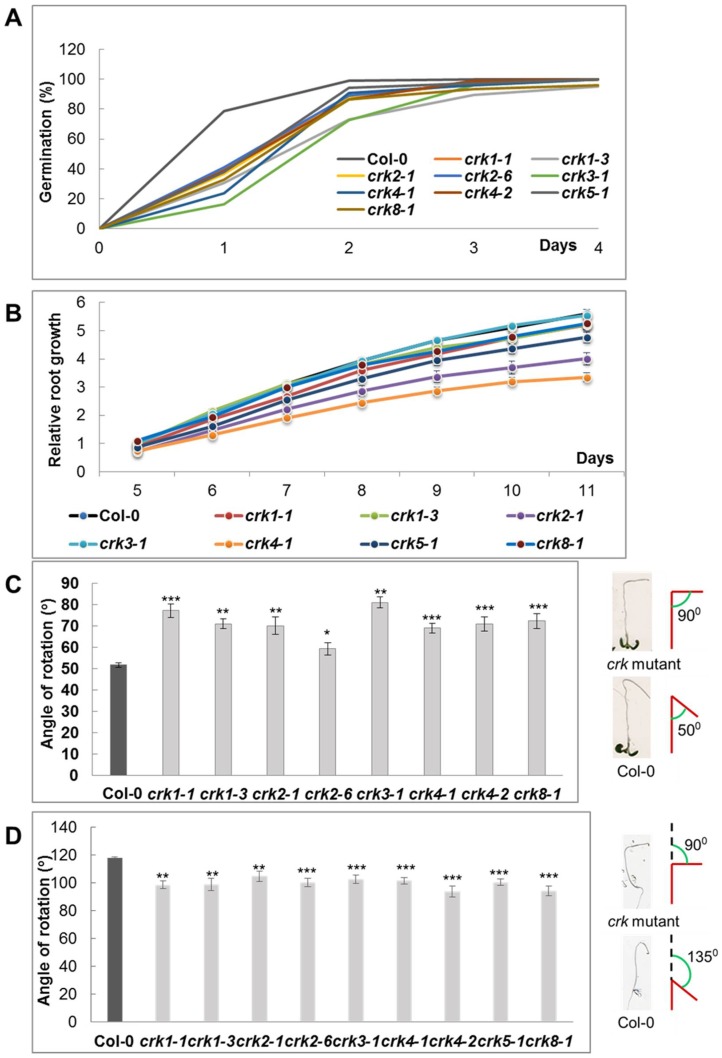
Germination and early seedling development of A*tcrk* mutants. (**A**) Germination of At*crk* mutants on standard half strength Murashige and Skoog (MS) medium [15]; (**B**) Root growth rate of seedlings grown on vertical plates for 14 days under continuous light conditions; (**C**) Root gravitropic test. Six days old, vertically grown seedlings were gravistimulated by changing orientation of plates by 135° and the angle of root bending was recorded 24 h after reorientation; (**D**) Hypocotyl gravitropic test. Seedlings were germinated in dark and plates were reoriented 5 days after germination. Hypocotyl angles were scored 24 h after; (**C**,**D**) Pictograms show the mode of root and hypocotyl bending measurements. Values are means ± SE. Statistically significant values were calculated by Student’s *t* test for * *p* < 0.05, ** *p* < 0.005 and *** *p* < 0.0005 (*n* = 30, two independent biological repetitions).

Phenotypic characterization of the mutants included series of assays in which germination, plant growth and gravitropic responses of the mutants were tested. Germination of the same age mutant seeds was compared by counting rates of cotyledon opening in short day conditions (Figure 4A). All At*crk* mutants had delayed germination. While 80% of wild type seeds germinated after one day of incubation in the culture room, 20% to 40% of the mutant seeds germinated by this time. Germination was, however, temporarily delayed, as nearly 100% of seeds germinated three days after sowing (Figure 4A).

Mutant seedlings were similar to wild type ones when grown on standard culture conditions, in short daylength. Inactivation of *AtCRK5* was earlier reported to inhibit root elongation and delay gravitropic bending of roots [15]. These characters were therefore tested in the other At*crk* mutants also. Root growth rates were investigated by measuring the root lengths at different time points for all At*crk* mutants under continuous light condition from 5th to 11th days after germination. Most of the At*crk* mutants showed reduced root elongation rate. The difference in root growth rate was significantly lower in At*crk2-1*, At*crk4-1* and At*crk5-1* mutants, while a slight but not significant reduction in growth rate was detected in At*crk1-1*, At*crk1-3,* and At*crk8-1* mutants. At*crk3-1* did not show any difference in the root elongation rate as compared to the wild type (Figure 4B).

Gravitropic response of the mutants was subsequently tested in root and hypocotyl bending assays as reported [15]. Comparing to the wild type, At*crk* mutants displayed a different degree of delayed root bending under continuous light (Figure 4C). Hypocotyl bending in dark-germinated seedlings was also delayed in all At*crk* mutants (Figure 4D). Defects in gravitropic response suggested, that in analogy to At*CRK5*, the other AtCRK genes may also be implicated in auxin transport regulation, which is essential for proper root or hypocotyl bending during gravitropic stimulation [15,30,31,32].

**Figure 5:**
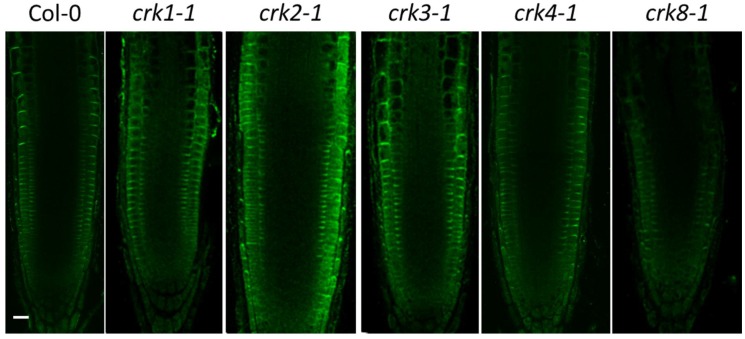
Immunolocalization of PIN2 in wild type Col-0 and the At*crk* mutants. PIN2 (green signal) shows normal localization in epidermal/cortex cells of Col-0 plants. PIN2 signal intensity or pattern is altered in most At*crk* mutants. Bar = 25 µm.

PIN-FORMED (PIN) proteins are known auxin transporters which regulate cell elongation [33]. From shoot tip, auxin is transported through the central vasculature toward root tip which is called as acropetal auxin transport carried out by Auxin transporter protein 1 (AUX1) influx carrier and PIN1, PIN4, PIN7 and ABCB1, ABCB19 efflux carriers [34]. In columella cells, the AUX1, PIN2 and PIN3 are the main auxin transporters responsible for basipetal auxin flow through lateral root caps and epidermis into the direction of elongation zone where the cell growth is regulated by the auxin distribution in *Arabidopsis* sp. roots [34]. PIN2 auxin efflux carrier has a characteristic two cell layer localization in the transient zone of the root epidermis and cortex cells [15,35]. Previously, an altered PIN2 localization pattern has been described in the At*crk5-1* mutant [15]. Therefore, localization of PIN2 was also tested in At*crk* mutants by immunolocalization. While PIN2 was properly distributed in epidermal and cortex cells of transition zone in wild type wild type Col-0 roots, aberrant PIN2 localization was observed in the root transient zone cells of each mutant, accompanied with altered PIN2 signal intensity or defective localization pattern (Figure 5).

The AtCRK5 phosphorylates the central hydrophilic T-loop of PIN2 in vitro, which is the part responsible for its plasma membrane (PM) association and stability [15]. Inactivation of AtCRK5 results in delayed root gravitropic response in At*crk5-1* [15]. We assume that inappropriate phosphorylation of PIN2 by At*crk* mutants may lead to the instability or enhanced degradation of the PIN2 protein, which could result in weak PIN2 signal intensities or improper localization pattern. In actuality, the At*crk* mutants displayed a delayed root gravitropic response upon gravistimulation.

### 2.4. AtCRK1 Is Implicated in Light Sensitivity

**Figure 6:**
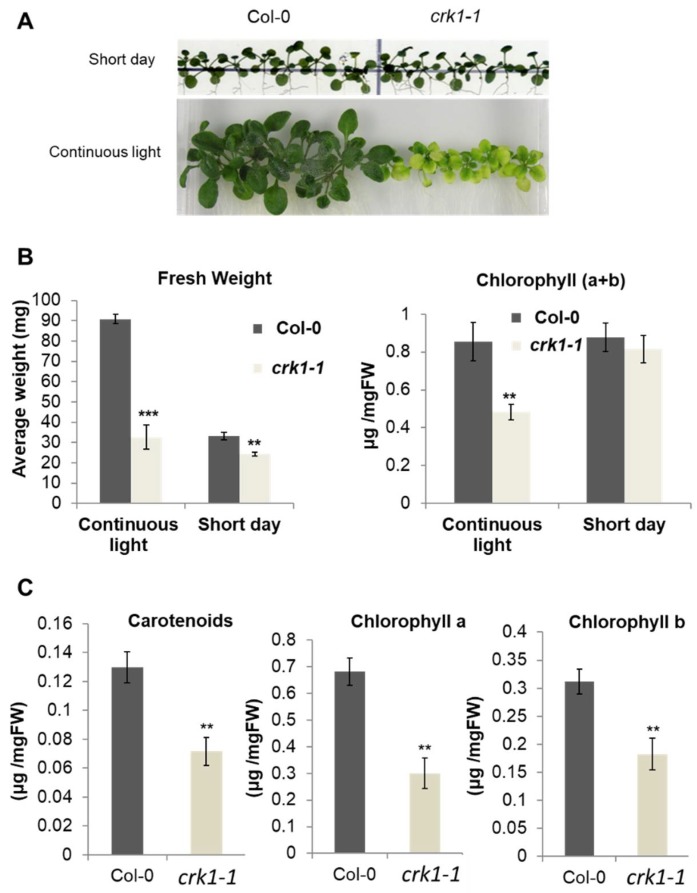
Light sensitivity of the At*crk1-1* mutant plants*.* (**A**) Images of 3-week old wild type (Col-0) and mutant (*crk1-1*) plantlets grown on vertical plates under continuous light or short day illumination; (**B**) Fresh weight and chlorophyll a+b contents of 3-week old plantlets under continuous light and short day conditions; (**C**) Carotenoid, chlorophyll a, chlorophyll b contents of 3-week old plantlets under continuous light. Values are means ± SD, *n* = 15 seedlings. Statistically significant values were calculated by Student’s *t* test for ** *p* < 0.005 and *** *p* < 0.0005.

Under short day illumination all At*crk* mutants were indistinguishable from wild type plants, whereas, after two/three weeks of continuous light illumination, only the At*crk1-1* mutant displayed semi-dwarf phenotype with serious growth defects (Figure 6A). When compared to the wild type, the rosette size of the At*crk1-1* plants was reduced, turning to a pale green coloration, indicating decreased chlorophyll content. In fact, the fresh weight of the At*crk1-1* plants was more than 60% smaller than the wild type ones under continuous light, while the difference in a short day was not more than 20% (Figure 6B). The chlorophyll a + b content of the At*crk1-1* mutant was also nearly 50% less than that of the wild type in continuous light, while there was no difference under a short day condition. When we checked the pigment content in continuous light, it was found that the chlorophyll a, chlorophyll b, and carotenoid contents of the At*crk1-1* mutant plants were around 50% lower than in wild type ones under continuous illumination (Figure 6C).

**Figure 7:**
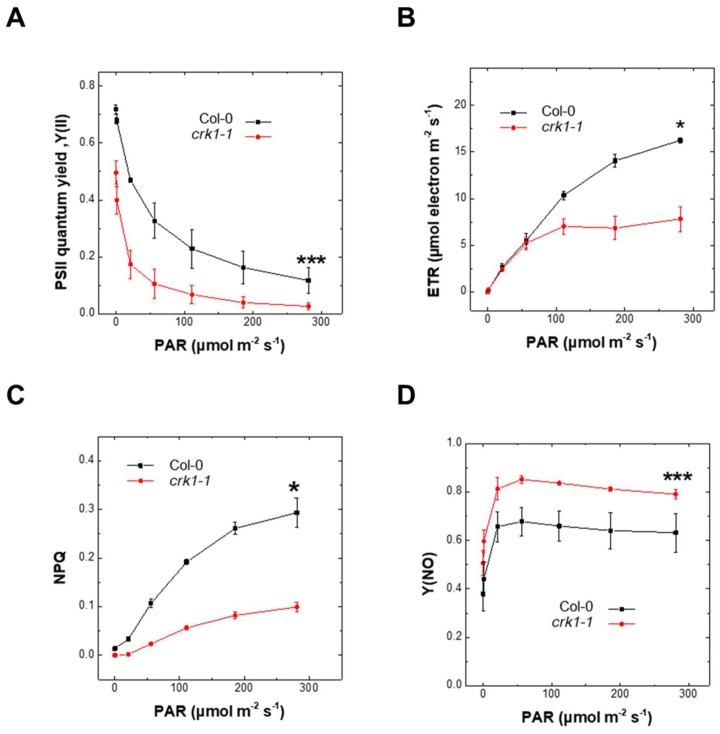
Photosynthetic parameters of At*crk1-1* mutant under continuous light. (**A**) Effective PSII quantum yield (Φ_PSII_). The At*crk1-1* mutant showed significantly lower effective PSII quantum yield values under all light intensities (*** *p* ≤ 0.001); (**B**) Electron transport rate (ETR). The At*crk1-1* mutant has significantly lower ETR values than wild type plants (* *p* ≤ 0.05); (**C**) Regulated Non- Photochemical Quenching (NPQ); (**D**) Non-regulated non-photochemical quenching (Y (NO)). The NPQ values of At*crk1-1* plants were decreased (* *p* ≤ 0.05), while the Y (NO) showed increased (*** *p* ≤ 0.001) values in At*crk1-1* mutant as compared to wild type plants. Data are presented as the mean ± standard error. A two-way ANOVA test was used for statistical analysis and was performed using the OriginPro 8.6 software (OriginLab Corporation, Northampton, MA, USA).

Reduced chlorophyll content can affect photosynthetic performance [36]. Therefore, we measured the photosynthetic efficiency of the At*crk1-1* mutant and wild type plants based on PSII chlorophyll fluorescence. PSII maximum quantum yield (*F*_v_*/F*_m_) represents the photosynthetic capacity and functional/structural integrity of PSII [36] and the *F*_v_*/F*_m_ values of the At*crk1-1* mutant were reduced when compared to Col-0 wild type plants (Figure 7A). The lower chlorophyll content of At*crk1-1* might directly be responsible for inferior F_v_/F_m_ values [37]. Under increasing photosynthetic active radiation (PAR), reduced PSII quantum yield, Φ_PSll_ values were detected in At*crk1-1* under all light intensities (Figure 7A). Electron transport rate (ETR) of the At*crk1-1* lagged behind the ETR of Col-0 wild type plants, especially at higher PAR (Figure 7B), indicating that adaptation capacity of mutant to higher light intensities is weaker. Values of regulated non-photochemical quenching (NPQ) in At*crk1-1* mutant were lower than that of wild type plants, while the non-regulated, non-photochemical quenching (Y (NO)) was higher (Figure 7C,D) [38]. These results suggest that the At*crk1-1* mutation results in defects of light harvesting capability and electron flow as well. Consequently, the efficiency of photosynthesis and excitation energy quenching ability is impaired in At*crk1-1* mutant. We did not find such alterations in the photosynthetic parameters of the other At*crk* mutants (Supplemental Figure S4).

**Figure 8:**
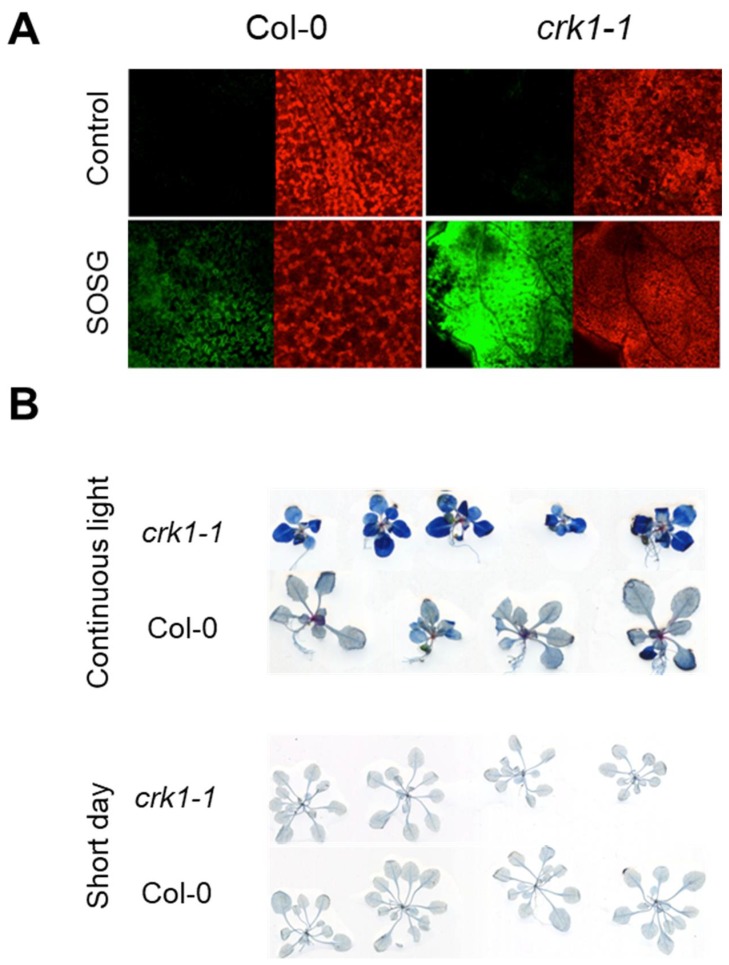
Reactive oxygen species (ROS) accumulation and cell death in At*crk1-1* mutant. (**A**) ^1^O_2_ production in leaves of At*crk1-1* mutant detected by fluorescence of the Singlet Oxygen Sensor Green (SOSG) probe. Plants were infiltrated with SOSG and exposed to high light (10 µM SOSG, 2300 µmole photons m^−2^ s^−1^ for 5 min). Fluorescence was detected by confocal laser scanning microscopy (CLSM). Note intensive fluorescence of SOSG-infiltrated mutant leaves; (**B**) Cell death in *Arabidopsis* sp. leaves, detected by Evans blue staining. 3-week old plants were grown either on short day or under continuous light. Note intensive Evans blue reaction in At*crk1-1* mutants when exposed to continuous light.

Reactive oxygen species (ROS) are by-products of photosynthetic processes, which are greatly affected by different stress conditions, leading to reduced photosynthetic performance [39,40,41]. Under excess light, the singlet oxygen (^1^O_2_) is the dominant ROS produced in the chloroplast, which is quite damaging and can lead to ROS- induced cell death [42,43,44]. Light sensitivity of At*crk1-1* prompted us to measure ROS accumulation, in particular singlet oxygen levels. ^1^O_2_ levels were estimated by fluorescence of the ^1^O_2_-specific Singlet Oxygen Sensor Green (SOSG) fluorescent probe in wild type and At*crk1-1* mutant after 5 min of high light treatment (2300 µmole photons m^−2^ s^−1^) in the presence of SOSG [45,46,47]. A considerable difference was observed in ^1^O_2_ production between the At*crk1-1* mutant and wild type Col-0 leaves (Figure 8A). Since ^1^O_2_ accumulation was higher in mutant leaves under continuous illumination, its deleterious effect was estimated by testing the cell death of wild type Col-0 and At*crk1-1* plants with Evans blue staining. Wild type Col-0 leaves retained much less dye than At*crk1-1* mutant leaves in continuous light but not under short day illumination, indicating an extensive cell death in At*crk1-1* leaves (Figure 8B). Differences in Evans blue staining confirmed that AtCRK1 may influence light-induced programmed cell death (PCD) in *Arabidopsis thaliana*.

## 3. Discussion

Protein phosphorylation is an essential regulatory process in signal transduction, which alters protein conformation, localization or stability. Protein kinases therefore play central role in such regulation and influence many developmental processes as well as responses to a wide variety of environmental or hormonal stimuli. Plant protein kinases are usually members of larger or smaller families, which are characterized by similar primary and secondary structure, but can be different in intracellular localization and substrate specificity. Transcriptional regulation of the encoding genes may also show considerable variability. Biological function of these genes can be partially overlapping, but can also be different Therefore characterization of one gene in a kinase family does not necessarily provide reliable functional information of the whole group.

The CDPK kinase family have been identified throughout the plant kingdom and represents a large calcium-sensing subfamily of serine/threonine protein kinases that have been identified only in the plant kingdom but not found in animals [1,5,7]. A significant amount of information is available on CDPKs, which are prominent regulators of plant development and responses to various abiotic and biotic stress conditions [2,3,5,7,48]. The CDPK-Related Kinase CRK family is closely related to CDPKs but—in contrast to CDPKs—only limited amount of information is available on their biological functions [2,4,13,15,23,49]. As a part of biochemical characterization of AtCRKs, it has been reported that tyrosine (Tyr) residues of β-tubulin (TBB) 2 and TBB7 have been phosphorylated by AtCRK2, AtCRK3, and AtCRK8 [27]. Consequently, the Tyr phosphorylation of endogenous TBBs decreased in At*crk2* and At*crk3* mutants. Tubulin is the major component of microtubules and protein-tyrosine kinase (PTK) and tyrosine-specific protein phosphatase (PTP) inhibitors are able to modify the stability and orientation of the microtubules [50,51]. Surprisingly, growth of At*crk2* or At*crk3* did not show significant difference as compared to wild type Col-0 plants under long day conditions [27]. By using a protein-protein interaction and in vitro kinase assays, four Tyr-phosphorylated transcription factors—ethylene response factor 13 (ERF13), WRKY DNA-binding protein 14 (WRKY14), ERF subfamily B-4 of ERF/AP2 transcription factor 2.6 (RAP2.6), and cryptochrome-interacting basic helix-loop-helix5 (CIB5)—have also been identified, which are substrates for AtCRK2 and AtCRK3 [27]. These results support the hypothesis that AtCRKs may be involved in protein tyrosine phosphorylation signaling in vivo [27]. Subcellular localization analyses indicated that AtCRK2 was mainly localized to the plasma membrane and partly to the nucleus. Since no plasma membrane-localized marker protein was used in this study, the possibility of cytoplasmic localization of AtCRK2 could not be excluded [27].

Our results show, besides the similarities, that there are certain differences in the biological function of the CRK gene family members. Similarly to AtCRK5, most AtCRK proteins showed plasma membrane localization either in protoplasts or in transgenic plants, overexpressing the CRK-eGFP fusions. Plasma membrane localization is in accordance with the N-terminal myristoylation sites, present in each CRK proteins, which is essential for membrane targeting [15,52]. Plasma membrane localization of all AtCRK-eGFP fusion proteins was confirmed by counterstaining with plasma membrane specific FM4-64 stain. Moreover, AtCRK1-GFP and AtCRK7-GFP displayed a peculiar localization pattern as their GFP-derived fluorescence pattern in root meristematic zone suggests that these AtCRKs might localize at the endomembrane system too. Such a possibility was confirmed by cell fractionation and detection of AtCRK1-GFP in both mitochondria/endomembrane and microsomal fractions. Similar localization has recently been described for an *Arabidopsis* sp. choline transporter-like1 (CTL1) protein, which regulates the secretory trafficking of auxin transporters PIN1 and PIN3 to control seedling growth [53].

Up to now there is limited information available on the biological function of the AtCRK kinase family. Analysis of T-DNA insertion mutants showed that, similar to the At*crk5-1* mutant, root geotropic responses of all At*crk* mutants was reduced when root and hypocotyl bending was analyzed. Altered PIN2 localization in all At*crk* mutants suggests that auxin transport is impaired in these mutants [15]. The PIN2 auxin efflux transporter is one of the most characteristic player of the basipetal auxin transport in *Arabidopsis* sp. roots [15,34,35,54]. We have reported that AtCRK5 phosphorylates the central hydrophilic T-loop of PIN2 in vitro, which controls its PM association and stability [15]. Inactivation of AtCRK5 results in deceleration of PIN2 exocytosis and as a result, it causes delayed root gravitropic response in At*crk5-1* mutant. In analogy to impaired phosphorylation of PIN2 in At*crk5-1*, altered immunolocalization pattern of this transporter in other At*crk* mutants can be the consequence of phosphorylation defects [15]. We assume that the inappropriate phosphorylation of PIN2 in the At*crk* mutants can lead to destabilization of the PIN2 protein, which may manifest in weak PIN2 signal intensities or improper localization pattern. Indeed, all the At*crk* mutants displayed delayed root gravitropic responses upon gravistimulation. Further studies are needed to decipher the precise function of PIN2 phosphorylation by individual AtCRKs.

Members of the kinase family might have different biological functions due to differences in transcriptional regulation, substrate specificity, intracellular localization, protein-protein interactions or both. While most mutants were similar to wild type plants in standard growth conditions, At*crk1-1* displayed light hypersensitivity under continuous illumination. AtCRK1 was previously implicated in the coordination of salt and heat stress responses. Enhanced salt sensitivity was reported to be accompanied by higher lipid peroxidation and inferior proline accumulation [23]. AtCRK1 kinase was reported to regulate heat shock signals by showing the interaction and phosphorylating the heat shock factor A1a (AtHSFA1a), and is the central regulator of responses to high temperature in *Arabidopsis* sp. While the At*crk1* T-DNA insertion mutant had impaired heat tolerance, overexpression of the AtCRK1 kinase improved it [25]. These results suggest that AtCRK1 is implicated in responses to different environmental stresses such as high temperature, extended light and salinity. Intracellular localization of AtCRK1 suggests that this kinase might be implicated in ER stress [55,56].

We have shown that singlet oxygen (^1^O_2_) content was higher in the At*crk1-1* mutant, indicating disturbances in photosynthetic electron transport under continuous light. Overreduction of the electron transport chain during light stress is known to generate ^1^O_2_, which can cause oxidative damage or generate stress signals. ^1^O_2_ generated by the reaction center chlorophyll of PSII can interact with and damage the D1 protein of the PSII reaction center that, following its oxidation, needs to be replaced by a newly synthesized D1 polypeptide [57]. Enhanced ^1^O_2_ content of At*crk1-1* was accompanied with low carotenoid and chlorophyll content, reduced PSII quantum yield (Y (II)), lower electron transport rate (ETR) and non-photochemical quenching (NPQ), suggesting that photosynthesis in this mutant is seriously impaired. The photosensible phenotype of At*crk1-1* is similar to the *Arabidopsis thaliana flu* and *chlorina (ch1)* mutants, which are characterized by high ^1^O_2_ content [44]. Similar to At*crk1-1*, the *Arabidopsis* mutants *flu* and *ch1* have large amounts of ^1^O_2_ without significant coproduction of other ROS such as hydrogen peroxide [44]. Light sensitivity of these mutants is accompanied with high lipid peroxidation rates as a result of ^1^O_2_-induced damage [43,44]. In addition, reduced NPQ is characteristic of both the At*crk1-1* and At*ch1* mutants [58]. Excessive ^1^O_2_ accumulation in *flu* mutant during dark-light transition generated necrotic lesions and activated a particular stress signaling pathway [59]. Necrotic lesions and cell death could be observed in the At*crk1-1* mutant in continuous light, which resembles the reaction of *flu* mutant from dark to light transition or *ch1* mutant to high light stress [44,59]. It is well documented that cell death can be the direct consequence of ^1^O_2_ hyper accumulation. ^1^O_2_ is not only toxic but it can also operate as a stress signal, leading to extensive changes in gene expression, promoting programmed cell death (PCD) or acclimation [43,44,59]. Similarity of the At*crk1-1* and At*ch1* mutants in light sensitivity, pigment composition, ROS generation, photoprotective NPQ and photosynthetic parameters suggest, that AtCRK1 is a suppressor of enhanced ^1^O_2_ production and can function as a regulator of ^1^O_2_-triggered cell death. Whether ^1^O_2_ generation is a result of decreased chlorophyll, carotenoid biosynthesis or another mechanism that damages the PSII reaction center remains to be elucidated by further experiments.

## 4. Materials and Methods

### 4.1. Plant Materials, Growth Conditions, Protoplast Transformation, Gravitropic Assays

In all cases, *Arabidopsis thaliana* (L.) Columbia-0 ecotype (Col-0) was used. Seeds were surface sterilized and kept at 4 °C for two days for stratification. Afterwards, seeds were transferred onto plates containing half-strength Murashige and Skoog medium (MS) with 0.5% sugar, 0.8% agar, pH: 5.7 [15]. The plates were placed vertically for root growth and gravitropic tests and incubated in thermostat room either under short day conditions (SD, 8h light/16 h dark cycle, 22 °C, 100 µmole photons m^−2^ s^−1^ light intensity) or under continuous light (CL, 22 °C, 50 µmole photons m^−2^ s^−1^ light intensity). The T-DNA insertion mutants for AtCRK1-AtCRK8 were acquired from the Salk Institute [60]: At*crk1-1* (SALK-071004), At*crk1-3* (SALK-037483), At*crk2-1* (SALK-080050), At*crk2-6* (SALK-090938), At*crk3-1* (SALK-142932), At*crk4-1*, (SALK-028536), At*crk4-2* (SALK-009503), At*crk8-1* (SALK-079502) [61,62]. The At*crk1-1* (SALK-071004) mutant was the same, which was used in earlier studies [23,25]. Localization of the T-DNA insertion of these mutants is shown in Figure S3. We could not confirm the presence of T-DNA insertions in *AtCRK6* and *AtCRK7* genes. The primers used for mapping T-DNA insertions and testing transcript levels by qRT-PCR are listed in Table S2. Mutants were genotyped and tested for gene expression level by qRT-PCR (Figure S3). According to transcript analysis, At*crk1-1,* At*crk2-1,* At*crk2-6,* At*crk3-1* and At*crk8-1* mutants were knockout (KO), while At*crk1-3*, At*crk4-1* and At*crk4-2* were knockdown (KD) mutants. Homozygous mutant lines were used for further analysis.

Transcript levels of AtCRK genes in different organs were tested in 14 days old wild type (Col-0) *Arabidopsis* sp. plants maintained in vitro at short day (SD) and continuous light (CL) conditions by qRT-PCR. Relative transcript levels were standardized to GAPDH-2 [63]. The measurements were performed with two biological replicates.

Intracellular localization of CRK-eGFP fusion protein was made either in protoplasts isolated from *Arabidopsis thaliana* suspension cultures or in transgenic plants expressing the 35S::cCRKs-GFP gene construct. Polyethylene glycol mediated (PEG) transformation of AtCRKs-eGFP constructs and transient expression studies were carried out as described [15,17]. To investigate the intracellular localization in plant roots, seven days old 35S::cCRKs-GFP seedlings grown vertically on ½ MS in short day condition (SD, 8h light/16h dark cycle, 22 °C, 100 µmole photons m^−2^ s^−1^ light intensity) were used*.*

Germination of the wild type Col-0 and mutant seeds of the same age was carried out on ½ MS medium in short day condition (SD, 8 h light/16h dark cycle, 22 °C, 100 µmole photons m^−2^ s^−1^ light intensity) and rates of cotyledon opening was scored during four days.

Root growth measurements were carried out on ½ MS medium under continuous light condition (CL, 22 °C, 50 µmole photons m^−2^ s^−1^ light intensity). Sterilized seeds were stratified for two days at 4 °C and seeds were put onto media kept horizontally. Four days after germination seedlings were transferred individually onto vertical plates and the end of root lengths were labelled every 24 h at the same time. Root growth rates of Col-0 and At*crk* mutants were measured from 5th day till 11th day and scanned seedling images were evaluated by ImageJ.

Root gravitropic assay was performed under continuous light in thermostat room as described (15). Hypocotyl bending assay was performed from seedlings germinated on vertical plates in dark for 6 days. Plates were subsequently rotated by 135° and bending angle were measured 24 h later by ImageJ. At least 50–50 wild type Col-0 and At*crk* mutant seedlings were tested for root/hypocotyl bending tests, in two independent experiments.

For fresh weight determination, rosettes of three seedlings were measured in five repeats. Two biological repetitions were performed. Chlorophyll and carotenoid contents were determined as described in [64]. Chlorophyll absorbance values were measured at 470, 648 and 664 nm between OD 0.3 and 0.8 using a Multiskan G0 spectrophotometer (Thermo Fischer Scientific, Vantaa, Finland). Chlorophyll concentrations were calculated with equations as described in [64].

### 4.2. Gene Cloning, Plant Transformation

Full length CRK cDNAs were PCR amplified from cDNA library with Phusion High-Fidelity DNA Polymerase (Thermo Fischer Scientific, Vilnius, Lithuania) using gene specific primers (Table S2) and cloned into pENTR2b Gateway vector. Error free cDNA clones were used to generate 35S::cCRKs-GFP gene fusions by cloning full length CRK cDNAs into pK7FWG2.0 expression vector [29] using with LR Clonase™ II enzyme (Thermo Fischer Scientific, Vilnius, Lithuania). Recovered clones were tested by sequencing and used for *Agrobacterium*-mediated transformation using the GV3101/pMP90 strain [65]. Col-0 wild type *Arabidopsis* sp. plants were transformed using in planta infiltration [66]. Primary transformants (T1) were selected on ½ MS medium containing 30 mg L^−1^ kanamycin. Plants of T3 generation homozygous lines were used for CRK-eGFP localization experiments.

### 4.3. Hairy Root Transformation of the 35S::cCRK1-GFP

The CD3-967/pBIN20-Golgi mCherry marker construct contains *Agrobacterium rhizogenes* strain (Arqua-1) was propagated on YEB agar plate supplemented with 100 mg L^−1^ kanamycin at 28 °C. For more information about this construct see reference [67]. Hairy root transformation was performed as described [68]. Briefly, *Arabidopsis thaliana* Col-0 plants expressing 35S::cCRK1-GFP construct were propagated on ½ MS media as indicated in Section 4.1. Five days old plants were used for transformation. CD3-967/pBIN20 containing Arqua-1 strain was inoculated into 10 mL liquid YEB supplemented with 100 mg L^−1^ kanamycin and rotated overnight at 28 °C in a shaker (200 rpm). A*grobacteria* were centrifuged and resuspend in ½ MS liquid medium. *Arabidopsis* sp. plants were immersed in this solution for 2–5 min. After two days co-incubation in dark, plants were transferred onto ½ MS agar plates with 200 mg L^−1^ cefotaxime (Duchefa, Haarlem, The Netherlands) and 200 mg L^−1^ carbenicillin (Duchefa, Haarlem, The Netherlands) and cultured at 22 °C. After three to four weeks, the newly formed hairy roots were used for microscopy studies.

### 4.4. Physiological Characterization of the Arabidopsis CRKs

#### 4.4.1. PSII Photochemical Activity Measurements

PSII Photochemical Activity Measurements were made on Col-0 wild type and At*crk* mutant plants, grown on vertical plates for two weeks as described above. Chlorophyll fluorescence was measured with MAXI-version of Imaging-PAM (Walz, Effeltrich, Germany). After dark adaptation for 15 min, minimum and maximum fluorescence yields were measured before and after a saturating pulse, respectively. The seedlings were exposed to seven intensities of photosynthetic active radiation (PAR) increasing from 0 to 281 µmole m^–2^ s^–1^. Each illuminating period was 10 s to ensure the minimum fluorescence in actinic light (F_s_) and was followed by an excitation pulse to yield the maximum fluorescence in actinic light (F_m_’). The PSII quantum yield Y (II) was calculated by the Imaging Win software as Y (II) = (*F*_m_’ − *F*_s_)/*F*_m_’ as described earlier by [69]. The kinetics of non-photochemical quenching (NPQ) were calculated as NPQ = (*F*_m_ − *F*_m_’)/*F*_m_’. Kinetics of PSII quantum yield Y (II), ETR and NPQ were obtained by averaging data from fifteen corresponding areas of interest in five different seedlings. The presented results are the average of three different biological repeats.

#### 4.4.2. Detection of ^1^O_2_ Production

Singlet oxygen (^1^O_2_) levels were detected by Singlet Oxygen Sensor Green (SOSG) reagent (Molecular Probes Inc., Eugene, OR, USA) in detached leaves of Col-0 wild type and At*crk1-1* mutant plants as described previously [45,46,47]. Seedlings were immersed in 250 μM SOSG (dissolved in water) and illuminated with 2300 µmole photons m^−2^ s^−1^ intensity visible light for 5 min. Leaf samples of infiltrated SOSG were fixed on cover slip with agar (0.8%). Imaging of green fluorescence was performed on Olympus FV1000 confocal microscope (Tokyo, Japan) using excitation at 504 nm and emission detection at 525 nm. Images were obtained with a transmitted light detection module with 488 nm excitation using a laser diode (LD). Simultaneously, chlorophyll fluorescence was also visualized using excitation by the 488 nm argon laser and fluorescence detection through a 650–750 nm filter.

#### 4.4.3. Determination of Cell Death

Cell death was estimated by Evans blue staining as described [70]. Three weeks old vertically grown plants were stained with 0.1% (*w/v*) Evans blue dye using vacuum infiltration. Then leaves were washed three times for 10 min with 0.05% Tween20 dissolved in 150 mM NaCl to remove unbound dye, while the chlorophyll was removed by 96% ethanol washing. Seedlings were photographed on white surface.

### 4.5. Microsomal Membrane Preparation by Differential Centrifugation and Immunoblotting

Differential centrifugation method was used to study CRK-GFP localization in transgenic seedlings expressing the 35S::cCRK1-GFP construct. The solutions and samples were kept on ice until use and all centrifugation process were performed at 4 °C. Five grams of fresh plant material were homogenized in 10 mL of ice cold extraction buffer (50 mM Tris-HCl pH.: 7.5, 1mM EDTA pH.:8.0, 350 mM sucrose, 1–2 mM PMSF, 1mM DTT, 1xPIC (P9599 Sigma Aldrich, St. Louis, MO, USA)). The solution was filtrated through Miracloth (Merck Millipore, Burlington, MA, USA,) and then it was centrifuged by 1000× *g* for 5 min to remove cell debris. Supernatant was collected into new centrifuge tubes. 200 µL supernatant was taken into a microcentrifuge tube on ice that represented the total extract (total). This extract was centrifuged with 5000× *g* for 10 min and supernatant was transferred into a new centrifuge tube. The pellet was resuspended in 1 mL extraction buffer and pipetted into 1.5 mL microcentrifuge tube. Using a precooled table top centrifuge, the pellet—representing plastids and nuclei at 5000 g—was collected again. 200 µL from the supernatant was also saved, this was the 5000 g supernatant (soluble A). After repeating this step at 15,000 g for 15 min, mitochondria, part of the endomembrane, and soluble B fractions were obtained. Finally, centrifugation with 48,000 g for one hour resulted in cytoplasmic and microsomal (all endomembrane and plasma membrane) fractions. Protein concentration was determined using Bradford reagent (Bio-Rad Laboratories, Hercules, CA, USA). 25 μg total protein from each sample was size separated on 8% SDS-PAGE, transferred onto Immobilon PVDF Membrane (Merck Millipore, Burlington, MA, USA), incubated 1 h in 1 × TBST blocking buffer (50 mM Tris-HCl (pH 8.0), 150 mM NaCl, 0.05% Tween-20, 5% dry skimmed milk) and 1.5 h with anti-GFP antibody (Roche, 1:2000 dilution) in blocking buffer. After washing with 1 × TBST three times for 10 min, the membranes were incubated for 1.5 h with an anti-mouse-POD secondary antibody (Pierce, dilution 1:5000), washed with 1 × TBST as before and then overlaid with Immobilon Western Chemiluminescent HRP Substrate (Merck Millipore, Burlington, MA, USA) to detect 35S::cCRK1-GFP by autoradiography.

In the case of 35S::cCRK1-GFP, 35S::cCRK2-GFP, 35S::cCRK3-GFP, 35S::cCRK4-GFP, 35S::cCRK5-GFP, 35S::cCRK7-GFP and 35S::cCRK8-GFP expressing plants, differential centrifugation was made as described [71]. At least 100 mg of frozen plant materials were ground in liquid nitrogen and then resuspended in ice cold (350 µL/100 mg fresh weight) extraction buffer (50 mM MOPS-KOH pH.:7.5, 5% glycerol, 810 mM sucrose, 10 mM EDTA pH.:8.0, 5 mM EGTA pH.:8.0, 1–2 mM PMSF, 1–2 × PIC—Sigma P9599, 1mM DTT). Samples were centrifuged by 600× *g* for 4 min to remove cell debris, then supernatant was filtrated through Miracloth (Merck Millipore, Burlington, MA, USA) filled blue tip, then centrifuge again (600× *g*, 4 min) to remove cell debris. Supernatant was collected into new microcentrifuge tube and diluted with sterile water (1:1). A 50 µL aliquot saved as a total extract (T) and the rest of the extract was centrifuged at 4 °C (21,130× *g* for 1.5 h in Eppendorf 5424 R centrifuge). Supernatant was taken into a new tube which represents the soluble fraction or cytoplasmic (Cy), while the pellet represents the microsomal (Mi) fraction. The microsomal pellet was washed with membrane wash buffer (50 mM Tris-HCl pH.:7.5, 5 mM EDTA pH.:8.0, 5 mM EGTA pH.:8.0, 1–2 × PIC—Sigma P9599, 1 mM DTT) and recentrifuged for 50 min at 21,130× *g* at 4 °C. Supernatant was discarded. The microsomal pellet and the supernatants from previous steps were stored in −80 °C until use. To detect the various AtCRK-GFP fusion proteins, a Western blot was performed as above.

### 4.6. RNA Isolation and Real Time Quantitative PCR

Total RNA was isolated from 100 mg *Arabidopsis* sp. leaves and roots and from whole seedlings for KO/KD expression studies using Nucleospin Plant RNA kit (Macherey-Nagel, Düren, Germany). First-strand cDNA synthesis of 1 μg of total RNA was carried out in 20 μL with RevertAid M-MuLV Reverse Transcriptase (Applied Biosystems by Thermo Fischer Scientific, Vilnius, Lithuania), using random hexamers. Real-time PCR was carried out with the ABI 7900 Fast Real Time System (Applied Biosystems, Foster City, CA, USA) with the following protocol: 45 cycles at 95 °C for 15 s, followed by 60 °C for 1 min. The normalized relative transcript levels were obtained by the 2^−ΔCt^ method [63]. At least two biological replicates were performed for each gene tested.

### 4.7. PIN2 Immunolocalization

PIN2 immunolocalization was basically performed with wild type Col-0 and At*crk* mutant seedlings grown vertically on ½ MS medium supplemented with 0.5% sucrose for 7 days in constant light as described in [15].

### 4.8. Microscopy

For CRK-eGFP localization studies, six days old seedlings expressing the 35S:CRKs-GFP constructs were imaged using Olympus FV1000 confocal laser scanning microscopy system with IX81 invert microscope and 60× oil N.A 1.35 objective (Tokyo, Japan). Excitation sources were 405 nm (root cell autofluorescence), 488 nm (eGFP) and 543 nm (FM4-64, Mito Tracker Orange, mCherry) lasers. FM4-64 (Invitrogen, Waltham, MA, USA) staining was carried out on 6 days old seedlings by incubating the samples in 5 µM FM4-64 for 10 min. Spinning disk confocal images were recorded using Visitron spinning disk confocal system (Visitron systems GmBH, Puchheim, Germany) equipped with Yokogawa CSU-W1 spinning disk unit (50 µm pinhole diameter), Olympus IX83 inverted microscope (60× oil objective, N.A 1.42), Andor Zyla 4.2 Plus camera, 488 and 561 nm lasers. Composite images were prepared using the Adobe Photoshop, Adobe Illustrator, (Adobe Systems Incorporated, San Jose, CA, USA) and Corel Photopaint (X7) (Ottawa, Canada) software.

### 4.9. Bioinformatics Analysis

Primers for genes investigated were constructed using Primer3Plus [72]. For DNA manipulation VectorNTI (Thermo Fisher Scientific, Waltham, MA, USA) and Lasergene (DNASTAR Inc., Madison, WI, USA) program suits were used. A phylogenetic tree was constructed by using MEGA5.05 software [73]. During phylogeny reconstruction, a neighbor-joining bootstrap method with 500 bootstrap replications was used.

### 4.10. Accession Numbers

Sequence data used in this study can be found in the Arabidopsis Information Resource (TAIR) and GenBank (NCBI) databases under the following accession numbers: *AtCRK1 (At2g41140)*, *AtCRK2 (At3g19100)*, *AtCRK3 (At2g46700)*, *AtCRK4 (At5g24430)*, *AtCRK5 (At3g50530)*, *AtCRK6 (At3g49370)*, *AtCRK7 (At3g56760)*, *AtCRK8 (At1g49580)*, *PIN2 (At5g57090*) and *GAPDH2 (At1g13440).*

## Supplementary Materials

Supplementary materials can be found at http://www.mdpi.com/1422-0067/19/5/1282/s1.
